# Periostin Mediates Right Ventricular Failure through Induction of Inducible Nitric Oxide Synthase Expression in Right Ventricular Fibroblasts from Monocrotaline-Induced Pulmonary Arterial Hypertensive Rats

**DOI:** 10.3390/ijms20010062

**Published:** 2018-12-24

**Authors:** Keisuke Imoto, Muneyoshi Okada, Hideyuki Yamawaki

**Affiliations:** Laboratory of Veterinary Pharmacology, School of Veterinary Medicine, Kitasato University, Higashi 23 bancho 35-1, Towada City, Aomori 034-8628, Japan; dv16001@st.kitasato-u.ac.jp (K.I.); yamawaki@st.kitasato-u.ac.jp (H.Y.)

**Keywords:** pulmonary arterial hypertension, monocrotaline, right ventricular failure, nitric oxide, inducible nitric oxide synthase, periostin, right ventricular fibroblasts, l-type Ca^2+^ channel

## Abstract

Pulmonary arterial hypertension (PAH) leads to lethal right ventricular failure (RVF). Periostin (*POSTN*) mRNA expression is increased in right ventricles (RVs) of monocrotaline (MCT)-induced PAH model rats. However, the pathophysiological role of POSTN in RVF has not been clarified. We investigated the effects of POSTN on inducible nitric oxide (NO) synthase (iNOS) expression and NO production, which causes cardiac dysfunction, in right ventricular fibroblasts (RVFbs). Male Wistar rats were intraperitoneally injected with MCT (60 mg/kg) or saline. Three weeks after injection, RVFbs were isolated from RVs of MCT- or saline-injected rats (MCT-RVFb or CONT-RVFb). In MCT-RVFb, iNOS expression and phosphorylation of extracellular signal-regulated kinase 1/2 (ERK1/2), c-Jun N-terminal kinase (JNK) and nuclear factor-kappa B (NF-κB) were higher than those in CONT-RVFb. Recombinant POSTN increased iNOS expression and NO production, which were prevented by a pharmacological inhibition of ERK1/2, JNK or NF-κB in RVFbs isolated from normal rats. Culture medium of POSTN-stimulated RVFbs suppressed Ca^2+^ inflow through l-type Ca^2+^ channel (LTCC) in H9c2 cardiomyoblasts. We demonstrated that POSTN enhances iNOS expression and subsequent NO production via ERK1/2, JNK, and NF-κB signaling pathways in RVFbs. POSTN might mediate RVF through the suppression of LTCC activity of cardiomyocytes by producing NO from RVFbs in PAH model rats.

## 1. Introduction

Pulmonary arterial hypertension (PAH) is characterized by a constriction of pulmonary artery by progressive vascular remodeling, which elevates pulmonary arterial resistance. For the management of PAH, pulmonary arterial vasodilators, such as endothelin receptor antagonist, phosphodiesterase-5 inhibitors and prostacyclin analogs, are widely used in recent two decades [1,2]. Although the vasodilators have effectively reduced mortality of PAH patients, it still remains high throughout the world [3,4,5]. PAH leads to right ventricular (RV) hypertrophy and subsequent lethal RV failure (RVF). Thus, a novel therapeutic strategy for PAH-induced RVF has been awaited. RV systolic dysfunction caused by RV diastolic stiffness worsens along with deterioration of RVF [6,7]. However, the mechanisms underlying initiation and progression of PAH-induced RVF have not been fully clarified.

Periostin (POSTN), a member of Fasciclin family, is an approximately 90 kDa secreted matricellular protein [8,9]. POSTN plays a role in embryogenesis, tumorigenesis, angiogenesis, and fibrosis by regulating various cellular functions including proliferation, migration, differentiation, and endothelial-mesenchymal transformation [10,11,12,13,14,15,16]. The biological functions of POSTN are mediated through the interaction between cell and extracellular matrix [16]. The expression of POSTN is increased in the heart tissue of myocardial infarction, cardiac hypertrophy and myocardial fibrosis [17,18,19]. It has been thought that POSTN promotes cardiac remodeling during the development of cardiac diseases via affecting functions of cardiac fibroblasts [19,20,21]. We have recently demonstrated that *POSTN* mRNA expression is increased in RVs of monocrotaline (MCT)-induced PAH model rats [22]. Moreover, phenotypical changes of RV fibroblasts (RVFbs) such as hyper-proliferation and migration in MCT-induced PAH model rats were observed [23]. However, the relationship between POSTN and phenotypical changes of RVFbs in the pathogenesis of PAH-induced RVF has not been elucidated.

Three different isoforms of nitric oxide (NO) synthase (NOS), neuronal NOS (nNOS), endotherial NOS (eNOS) and inducible NOS (iNOS), are known. In the heart tissue of various cardiovascular diseases including left ventricular hypertrophy, myocardial infarction and heart failure, the expression of iNOS is increased [24,25,26,27]. A knockdown of *iNOS* gene is protective against systolic dysfunction in pressure-overload-induced left ventricular hypertrophy and heart failure in mice [26,27]. iNOS and subsequent NO production play a role in inflammatory cytokines-induced systolic dysfunction in hearts [28]. In human chondrocytes, POSTN induces *iNOS* gene expression via nuclear factor-kappa B (NF-κB) [29]. In the present study, we investigated whether POSTN increases iNOS expression and subsequent NO production in RVFbs isolated from MCT-induced PAH model rats. Furthermore, we explored the effects of POSTN-induced NO from RVFbs on l-type Ca^2+^ channel (LTCC) activity in H9c2 cardiomyoblasts to clarify the pathophysiological role of POSTN in RVF of PAH.

## 2. Results

### 2.1. Expression of POSTN Was Increased in RVs of MCT-Injected Rats

**Figure 1:**
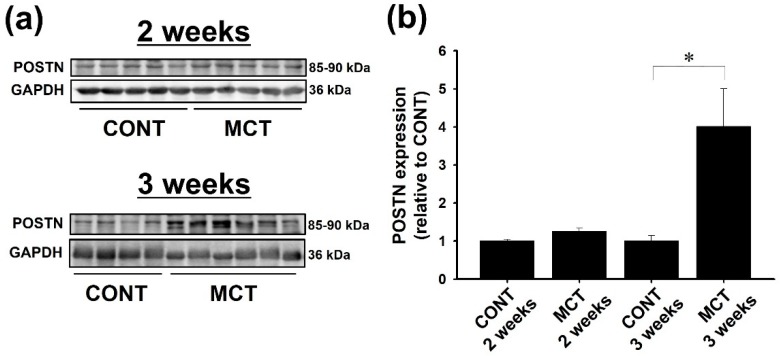

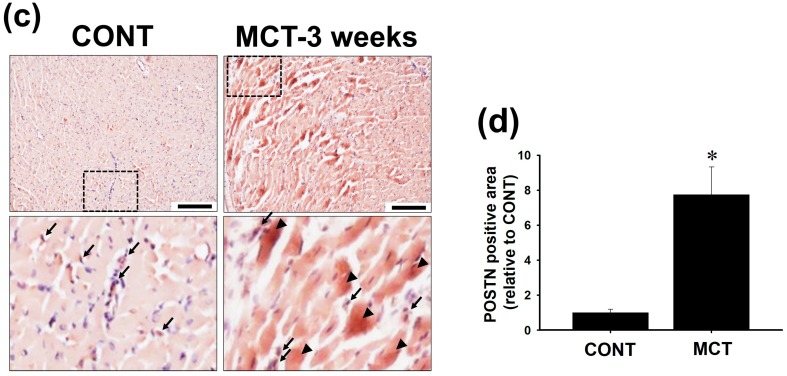
Protein expression of periostin (POSTN) was increased in right ventricles (RVs) of monocrotaline (MCT, 3 weeks)-injected rats. Expression of POSTN in RV tissues of MCT (2 weeks: *n* = 5; 3 weeks: *n* = 6)- or saline-injected (CONT, 2 weeks: *n* = 5; 3 weeks: *n* = 4) rats was evaluated by western blotting. (**a**) Representative blots were shown (upper: 2 weeks; lower: 3 weeks). (**b**) POSTN expression level was corrected by glyceraldehyde-3-phosphate dehydrogenase (GAPDH), and the normalized expressions relative to CONT were shown as mean ± standard error of the mean (S.E.M.). (**c**) The expression and distribution of POSTN in RVs of CONT and MCT (3 weeks)-injected rats was evaluated by immunohistochemical staining. Representative images for RVs from CONT (left) and MCT (3 weeks)-injected (right) rats were shown. Enlarged views of boxed regions in the upper original images were shown (3.8-fold magnification). Arrows indicate POSTN-positive interstitial cells. Arrowheads indicate POSTN-positive cardiomyocytes. (**d**) The ratio of POSTN-positive area to total RV area was calculated, and the normalized ratio relative to CONT was shown as mean ± S.E.M. (*n* = 3). Scale: 100 μm. * *p* < 0.05 versus CONT.

The protein expression of POSTN in RVs of MCT (3 weeks)-injected rats but not MCT (2 weeks)-injected rats was significantly higher than saline-injected (CONT) rats (Figure 1a,b, CONT: *n* = 4; MCT: *n* = 6, *p* < 0.05). Although POSTN is mainly expressed in fibroblast, the gene expression of *POSTN* is also detected in cardiomyocytes [30]. In immunohistochemical staining, the expression of POSTN was observed in the interstitial cells in RVs of CONT rats. On the other hand, the expression of POSTN was strongly increased in cardiomyocytes in RVs of MCT (3 weeks)-injected rats (Figure 1c, *n* = 3, *p* < 0.05). Of note, hypertrophy of cardiomyocytes was observed in RVs of MCT (3 weeks)-injected rats possibly due to PAH-induced pressure-overload (Figure 1c).

### 2.2. Expression of iNOS and Phosphorylation of Extracellular sSignal-Regulated Kinase 1/2 (ERK1/2), c-Jun N-Terminal Kinase (JNK) and NF-κB Were Enhanced in MCT-RVFb

**Figure 2:**
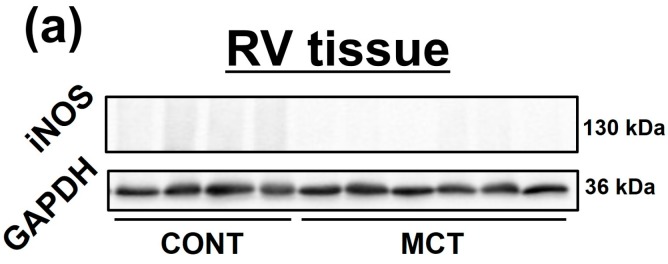

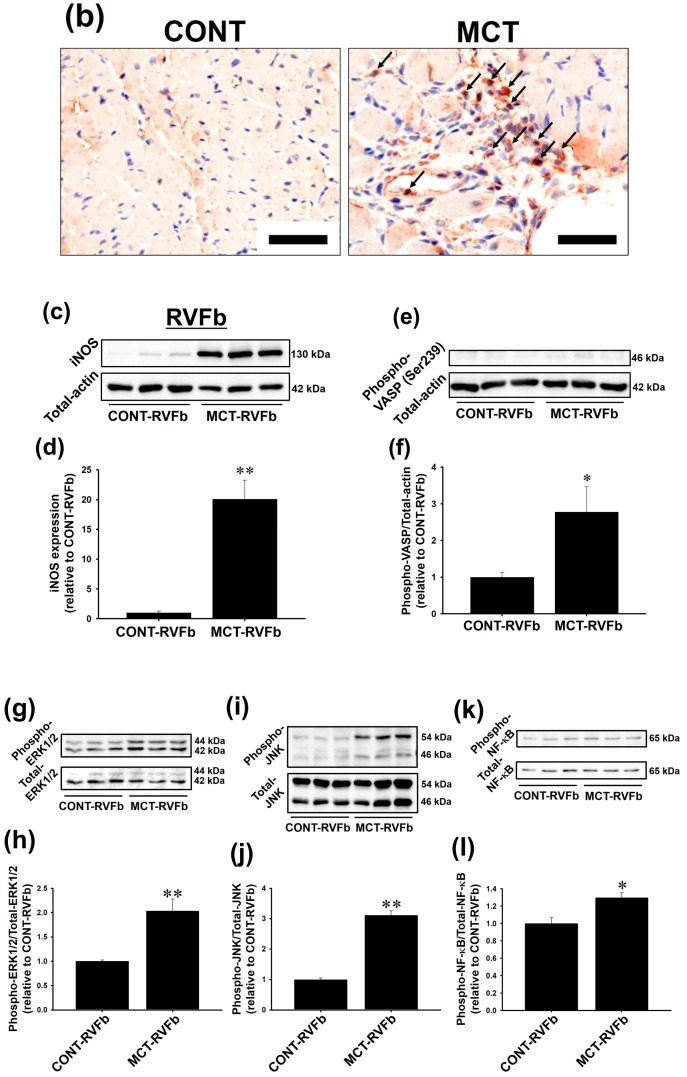
Protein expression of inducible nitric oxide (NO) synthase (iNOS) and phosphorylation of vasodilator-stimulated phosphoprotein (VASP), extracellular signal-regulated protein kinase 1/2 (ERK1/2), c-Jun N-terminal kinase (JNK) and nuclear factor-kappa B (NF-κB) were enhanced in RV fibroblasts (RVFbs) isolated from RVs of MCT-injected rats (MCT-RVFb). The expression of iNOS in RVs of CONT and MCT (3 weeks)-injected rats and in RVFbs isolated from the RVs of CONT rats (CONT-RVFb) and MCT-RVFb was evaluated by western blotting. (**a**) Representative blots in RVs were shown (CONT: *n* = 4; MCT: *n* = 6). (**b**) The distribution of iNOS in RVs was evaluated by immunohistochemical staining. Representative images for RVs from CONT (left) and MCT (3 weeks)-injected (right) rats were shown (CONT: *n* = 4; MCT: *n* = 6). Arrows indicate iNOS strongly positive interstitial cells. Scale: 50 μm. (**c**) Representative blots in CONT-RVFb and MCT-RVFb were shown. (**d**) iNOS expression levels were corrected by total-actin, and the normalized expressions relative to CONT-RVFb were shown as mean ± S.E.M. (*n* = 6). ** *p* < 0.01 versus CONT-RVFb. (**e**–**l**) Phosphorylation level of VASP, ERK1/2, JNK and NF-κB in CONT-RVFb or MCT-RVFb was evaluated by western blotting. (**e**,**g**,**i**,**k**) Representative blots were shown. (**f**,**h**,**j**,**l**) Phosphorylation level of VASP or ERK1/2, JNK and NF-κB was corrected by total-actin or total protein expression, and the normalized phosphorylation relative to CONT-RVFb was shown as mean ± S.E.M. (*n* = 6). *, ** *p* < 0.05, 0.01 versus CONT-RVFb.

The protein expression of iNOS was undetectable in whole RV tissues by western blotting (Figure 2a, CONT: *n* = 4; MCT: *n* = 6). In immunohistochemical staining, the interstitial cells in RVs of CONT rats were scarcely positive for iNOS. On the other hand, iNOS expression in the interstitial cells in RVs of MCT (3 weeks)-injected rats was increased. The cardiomyocytes in both RVs of CONT and MCT-injected rats were scarcely positive for iNOS (Figure 2b, CONT: *n* = 4; MCT: *n* = 6). The expression level of iNOS and phosphorylation level of vasodilator-stimulated phosphoprotein (VASP), a downstream molecule for NO/cyclic GMP (cGMP)-dependent signaling pathway, in RVFbs isolated from MCT (3 weeks)-injected rats (MCT-RVFb) were significantly higher than those in RVFbs isolated from CONT rats (CONT-RVFb) (Figure 2c,d, *n* = 6, *p* < 0.01; Figure 2e,f, *n* = 6, *p* < 0.05). The phosphorylation level of ERK1/2, JNK and NF-κB in MCT-RVFb was significantly higher than that in CONT-RVFb (Figure 2g–j, *n* = 6, *p* < 0.01; Figure 2k,l, *n* = 6, *p* < 0.05).

### 2.3. Recombinant Rat POSTN Increased Phosphorylation of ERK1/2, JNK and NF-κB in RVFbs

**Figure 3:**
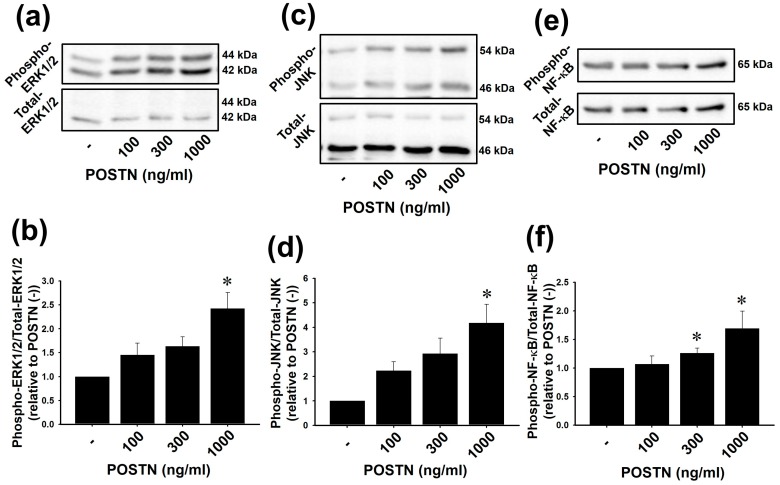
Recombinant rat POSTN activated ERK1/2, JNK and NF-κB in RVFbs isolated from RVs of non-treated normal rats. RVFbs were stimulated with recombinant rat POSTN (100–1000 ng/mL) or solvent (POSTN (-)) for 1 h. Phosphorylation of ERK1/2, JNK and NF-κB in RVFbs was evaluated by western blotting. (**a**,**c**,**e**) Representative blots were shown. (**b**,**d**,**f**) Phosphorylation level of ERK1/2, JNK and NF-κB was corrected by the total protein, and the normalized phosphorylation relative to POSTN (-) was shown as mean ± S.E.M. (*n* = 3). * *p* < 0.05 versus POSTN (-).

We next investigated whether the increased expression of POSTN is responsible for the activation of ERK1/2, JNK and NF-κB and induction of iNOS in MCT-RVFb. Recombinant rat POSTN-stimulation in non-treated normal RVFbs was performed to clarify it. POSTN (100–1000 ng/mL, 1 h) significantly increased phosphorylation of ERK1/2, JNK and NF-κB in RVFbs in a concentration-dependent manner (Figure 3a–f, *n* = 3). The maximum effect of POSTN was observed at 1000 ng/mL (Figure 3a–f, *n* = 3, *p* < 0.05). Thus, we used 1000 ng/mL of POSTN in the following experiments.

### 2.4. Recombinant Rat POSTN Induced iNOS Expression and NO Production in RVFbs

**Figure 4:**
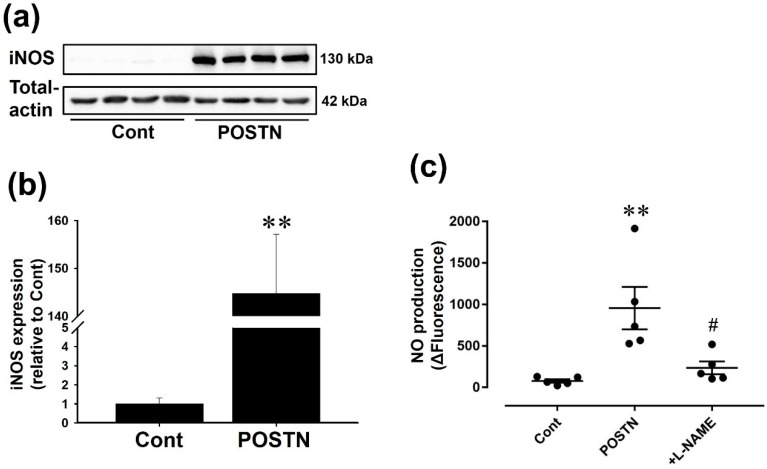
Recombinant rat POSTN enhanced iNOS protein expression and NO production in RVFbs. RVFbs were stimulated with recombinant rat POSTN (1000 ng/mL) or solvent (Cont) for 24 h. iNOS expression in RVFbs was evaluated by western blotting. (**a**) Representative blots were shown. (**b**) Expression level of iNOS was corrected by total-actin, and the normalized expression relative to Cont was shown as mean ± S.E.M. (*n* = 4). NO production was measured by using an NO-specific fluorescent dye 4,5-diamino-fluorescein (DAF-2). (**c**) The fluorescence at 120 min after treatment with l-arginine in the presence (+l-NAME) or absence of pretreatment with *N* (ω)-nitro-l-arginine methyl ester (l-NAME, 600 μM, 10 min), was subtracted by the fluorescence at 0 min (ΔFluorescence), and shown as mean ± S.E.M. (*n* = 5). ** *p* < 0.01 versus Cont, ^#^ *p* < 0.05 versus POSTN.

Recombinant rat POSTN (1000 ng/mL, 24 h) significantly increased iNOS protein expression (Figure 4a,b, *n* = 4, *p* < 0.01) and NO production (Figure 4c, *n* = 5, *p* < 0.01) in RVFbs. The enhanced NO production was significantly inhibited by *N* (ω)-nitro-l-arginine methyl ester (l-NAME, 600 μM, 10 min pre-treatment), an inhibitor of NOS (Figure 4c, *n* = 5, *p* < 0.05).

### 2.5. Pharmacological Inhibition of NF-κB, ERK1/2 or JNK Suppressed the POSTN-Induced iNOS Expression and NO Production in RVFbs

**Figure 5:**
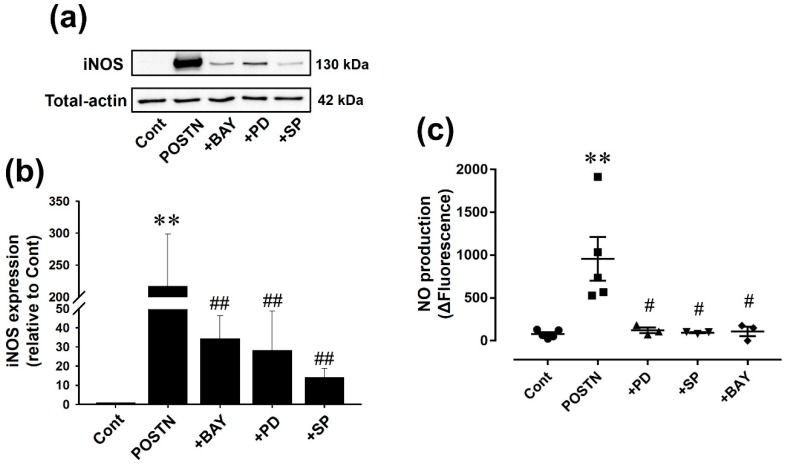
Pharmacological inhibition of inhibitor of kappa Bα (IκBα), mitogen-activated protein kinase kinase (MEK)/ERK signaling pathway or JNK prevented POSTN-induced iNOS expression and NO production in RVFbs. RVFbs were stimulated with recombinant rat POSTN (1000 ng/mL) or solvent (Cont) for 24 h in the presence or absence of pretreatment with inhibitor of ΙκΒα (BAY11-7082 (+BAY, 10 μM)), MEK/ERK signaling pathway (PD98059 (+PD, 50 μM)) or JNK (SP600125 (+SP, 10 μM)) for 30 min. The iNOS expression in RVFbs was evaluated by western blotting. (**a**) Representative blots were shown. (**b**) Expression level of iNOS was corrected by total-actin, and the normalized expression relative to Cont was shown as mean ± S.E.M. (*n* = 3). NO production was measured by using DAF-2. (**c**) The fluorescence at 120 min after treatment with l-arginine was subtracted by fluorescence at 0 min (ΔFluorescence), and shown as mean ± S.E.M. (Cont, POSTN: *n* = 5; +BAY, +PD, +SP: *n* = 3). ** *p* < 0.01 versus Cont, ^#^, ^##^ *p* < 0.05, 0.01 versus POSTN.

Pharmacological inhibition of signaling pathways with BAY11-7082, an inhibitor of kappa Bα (IκBα)/NF-κB inhibitor, PD98059, a mitogen-activated protein kinase kinase/ERK signaling pathway inhibitor, or SP600125, a JNK inhibitor, suppressed POTSN-induced iNOS expression (Figure 5a,b, *n* = 3, *p* < 0.01) and NO production (Figure 5c, Cont, POSTN: *n* = 5; +BAY, +PD, +SP: *n* = 3, *p* < 0.05) in RVFbs.

### 2.6. Culture Media (CM) Derived from POSTN-Stimulated RVFbs Inhibited LTCC Activity of H9c2 Cardiomyoblasts

**Figure 6:**
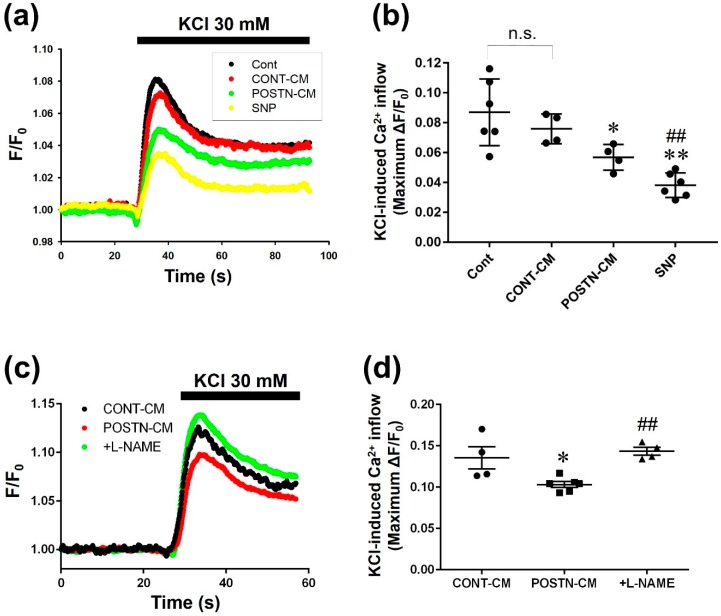
Culture media (CM) derived from POSTN-stimulated RVFbs inhibited Ca^2+^ inflow through l-type Ca^2+^ channel (LTCC) in H9c2 cardiomyoblasts. RVFbs were stimulated for 24 h with recombinant rat POSTN (1000 ng/mL) or solvent (CONT). Then, the RVFbs were incubated in new serum-free Dulbecco Modified Eagle’s Medium containing l-arginine (1 mM) for 24 h in the presence or absence of pretreatment with l-NAME (600 μM, 10 min). CM derived from solvent-stimulated RVFbs (CONT-CM) and from POSTN-stimulated RVFbs in the presence (+l-NAME) or absence (POSTN-CM) of l-NAME were collected. H9c2 cardiomyoblasts grown to confluent on a coverslip were treated with the CM. Ca^2+^ inflow through LTCC was measured by a fluorescent calcium measuring system using Fura-2 AM, a fluorescent calcium indicator. F340/F380 ratio (F), was calculated and normalized by the basal fluorescence (F_0_) at 30 seconds before KCl (30 mM)-stimulation (F/F_0_). (**a**) Average time course of F/F_0_ in H9c2 cardiomyoblasts treated for 1 h with milliQ (Cont), CONT-CM, POSTN-CM or sodium nitroprusside (SNP, 100 μM). The fluorescence was recorded every 0.1 s. (**b**) The maximum F/F_0_ change (ΔF/F_0_) by KCl-induced Ca^2+^ inflow was shown as mean ± S.E.M. (Cont, SNP: *n* = 6; CONT-CM, POSTN-CM: *n* = 4). n.s.: not significant, *, ** *p* < 0.05, 0.01 versus Cont, ^##^ *p*<0.01 versus CONT-CM. (**c**) The effect of pretreatment with l-NAME on average time course of F/F_0_ in H9c2 cardiomyoblasts treated for 1 h with POSTN-CM. (**d**) The maximum F/F_0_ change (ΔF/F_0_) by KCl-induced Ca^2+^ inflow was shown as mean ± S.E.M. (CONT-CM, +l-NAME: *n* = 4; POSTN-CM: *n* = 6). * *p* < 0.05 versus CONT-CM, ^##^ *p* < 0.01 versus POSTN-CM.

It has been reported that NO regulates LTCC activity in cardiomyocytes [31,32]. We confirmed that sodium nitroprusside (SNP, 100 μM), an NO donor, inhibited the KCl-induced Ca^2+^ inflow, which was caused by LTCC activation, in H9c2 cardiomyoblasts (Figure 6a,b, Cont, SNP: *n* = 6, *p* < 0.01). The CM from POSTN-stimulated RVFbs (POSTN-CM) but not from solvent-stimulated RVFbs (CONT-CM) significantly inhibited the KCl-induced Ca^2+^ inflow (Figure 6a,b, Cont: *n* = 6; CONT-CM, POSTN-CM: *n* = 4, *p* < 0.05). Furthermore, the inhibition of LTCCs by POSTN was disappeared by the pretreatment with l-NAME (600 μM, 10 min pre-treatment) (Figure 6c,d, CONT-CM, +l-NAME: *n* = 4; POSTN-CM: *n* = 6, *p* < 0.01).

## 3. Discussion

The present study for the first time clarified that POSTN, expression of which is increased in RVs of MCT-induced PAH model rats, induces iNOS protein expression and subsequent NO production via activating ERK1/2, JNK and NF-κB signaling pathways in RVFbs. Furthermore, the NO-containing CM derived from POSTN-stimulated RVFbs inhibited LTCC activity in H9c2 cardiomyoblasts.

We previously demonstrated that the mRNA expression of *POSTN* is increased in the RVs of MCT (2 and 3 weeks)-injected rats [22]. In the present study, we showed that the protein expression of POSTN in RVs was increased in MCT (3 weeks)-injected but not MCT (2 weeks)-injected rats (Figure 1a). Human and mouse POSTNs are degraded into 7 and 35 kDa fragments by cathepsin K [33,34,35], which is increased in the pressure-overload-induced hypertrophied heart [36]. Therefore, it is proposed that the increase in POSTN protein expression in the RVs of MCT (2 weeks)-injected rats was blunted by the cathepsin K-dependent degradation. On the other hand, we speculate that the protein expression of POSTN was significantly increased in the RVs of MCT (3 weeks)-injected rats, possibly because the increase in *POSTN* mRNA expression in the RVs of MCT (3 weeks)-rats was much higher than that in the RVs of MCT (2 weeks)-injected rats [22]. POSTN, which is associated with the development of cardiac fibrosis [17,19], is known to promote the phenotypical change of fibroblasts into myofibroblasts with the increase of α-smooth muscle actin (α-SMA) expression and collagen type I production [10,37,38,39]. In contrast, we previously demonstrated that the phenotype of RVFbs isolated from MCT-injected rats is entirely different from that of myofibroblasts [23]. In the RVFbs, the proliferation, migration and matrix metalloproteinase-9 expression were increased, but the expression of α-SMA and collagen type I was decreased [23]. It is thus suggested that POSTN might be involved in the PAH-induced RVF of MCT-injected rats through the regulation of RVFbs functions other than the induction of differentiation into myofibroblasts.

Although the expression of iNOS in whole RV tissues was undetectable by western blotting, immunohistochemical staining showed that the iNOS expression in interstitial cells but not in cardiomyocytes was increased in RVs of MCT-injected rats compared with CONT rats in this study. Cardiac fibroblasts account for a large portion of interstitial cells in the heart tissue [40]. We confirmed that the expression of iNOS and phosphorylation of VASP, a downstream molecule for NO/cGMP-dependent signaling pathway, in MCT-RVFb was significantly higher than those in CONT-RVFb (Figure 2c–f). Thus, the increase of iNOS protein expression and subsequent NO production in RVFbs might be at least one possible mechanism underlying RVF in MCT-injected rats. The gene expression of *iNOS* is regulated by activator protein (AP)-1, a transcription factor downstream of mitogen-activated protein kinase signaling pathway, in addition to NF-κB [41,42,43]. POSTN induces *iNOS* gene expression via NF-κB in human chondrocytes [29]. In the present study, the phosphorylation level of ERK1/2, JNK and NF-κB was significantly enhanced in MCT-RVFb compared with CONT-RVFb (Figure 2g–l). Recombinant POSTN-stimulation increased the phosphorylation of ERK1/2, JNK and NF-κB in a concentration-dependent manner in RVFbs isolated from normal rats (Figure 3). Furthermore, recombinant POSTN-stimulation also induced iNOS expression and NO production in RVFbs, which were inhibited by a pharmacological inhibition of NF-κB, ERK1/2 or JNK (Figure 4 and Figure 5). It is thus suggested that the increased iNOS expression in MCT-RVFb might be induced by POSTN via activation of NF-κB, ERK1/2 and JNK signaling pathways. The pharmacological inhibition of ERK or JNK signaling pathway had no influence on the POSTN-induced activation of NF-κB (Figure S1). Thus, POSTN might induce the iNOS expression via activating NF-κB and AP-1 through ERK1/2 and/or JNK signaling pathway, independently. In addition, we clarified that POSTN significantly increased the expression of IL-1β in RVFbs (Figure S2). Therefore, POSTN might induce iNOS expression throught the production of IL-1β in RVFbs. POSTN is known to interact with integrins such as α_v_β_3_ and α_v_β_5_ on cell surface [44]. In keratinocytes, POSTN induces thymic stromal lymphopoietin expression via activating α_v_β_3_ integrin/NF-κB signaling pathway [45]. In dermal fibroblasts, POSTN activates cell proliferation via activating integrin/ERK1/2 signaling pathway [46]. POSTN also activates migration and invasion of renal carcinoma via integrin/focal adhesion kinase/JNK signaling pathway [47]. From these observations, POSTN might activate NF-κB, ERK1/2 and JNK signaling pathways by binding to integrins in RVFbs. Further study is needed to clarify the receptors for POSTN.

Cardiac dysfunction in PAH-induced RVF is partly caused by dysregulation of contraction-associated proteins, such as LTCC or titin via β-adrenergic receptor desensitization [2]. In ferret RV cardiomyocytes, NO inhibits LTCC via cGMP-dependent signaling pathway [31]. In the present study, we also confirmed that SNP, an NO donor, suppressed the KCl-induced Ca^2+^ inflow, which was caused by LTCC activation, in H9c2 rat cardiomyoblasts (Figure 6a,b). Thus, NO might inhibit LTCC activity in cardiomyocytes of rats. POSTN-CM but not CONT-CM suppressed the KCl-induced Ca^2+^ inflow in H9c2 cardiomyoblasts, which was inhibited by l-NAME, a NOS inhibitor (Figure 6). From these observations, NO derived from RVFbs might be involved in the cardiac dysfunction through the suppression of LTCC activity in cardiomyocytes during RVF. The activation of cGMP-dependent signaling pathway by NO donors inhibits voltage-gated Ca^2+^ channels including LTCC [31,48]. NO also inhibits LTCC activity via direct action by *S*-nitrosylation [48,49]. Thus, it is suggested that the inhibitory effects of POSTN-CM on LTCC activity might be mediated by NO/cGMP-dependent signaling pathway or *S*-nitrocylation of LTCC. Further study is needed to clarify the detailed inhibitory mechanisms of POSTN-induced NO from RVFbs on LTCC activity in RV cardiomyocytes.

## 4. Materials and Methods

### 4.1. Reagents and Antibodies

Reagent sources were as follows: MCT (Wako Pure Chemical Industries, Ltd., Osaka, Japan), l-NAME (Dojindo, Kumamoto, Japan), BAY11-7082 (Merck (Calbiochem), Darmstadt, Germany), PD98059 (Wako Pure Chemical Industries, Ltd.), SP600125 (Jena Bioscience, Jena, Germany) and SNP (Sigma Aldrich, St., Louis, MO, USA).

Antibody sources were as follows: anti-POSTN (1:100 dilution) (Proteintech, Rosemont, IL, USA), anti-glyceraldehyde-3-phosphate dehydrogenase (1:1000 dilution) (GeneTex, Irvine, CA, USA), anti-iNOS (1:250 dilution for western blotting or 1:100 dilution for immunohistochemistry) (Becton, Dickinson and Company, Franklin Lakes, NJ, USA or Bioss, Woburn, MA, USA), anti-total-actin (1:1000 dilution) (Sigma Aldrich), anti-phospho-VASP (1:500 dilution) (Abcam, Cambridge, UK), anti-phospho-ERK1/2 (1:1000 dilution), anti-phospho-JNK (1:250 dilution), anti-total-JNK (1:500 dilution), anti-phospho-NF-κB p65 (1:500 dilution) (Cell Signaling Technology, Madison, WI, USA), anti-total-ERK1/2 (1:100 or 1:200 dilution) (Santa Cruz Biotech, Santa Cruz, CA, USA or Bioss), anti-total-NF-κB p65 (1:500 dilution) (Santa Cruz Biotech), horseradish peroxidase (HRP)-conjugated anti-rabbit IgG and HRP-conjugated anti-mouse IgG (1:10,000 dilution) (Amersham Biosciences, Buckinghamshire, UK or Cell Signaling Technology).

### 4.2. Production of Recombinant Rat POSTN Protein in E. coli

All the experiments of gene recombination were approved by the President of Kitasato University (Approval number 3918 (10 July 2017)). The gene of full-length rat *POSTN* (Accession number: KM117173.1) fused to 6× Histidine (His-tag) was inserted into pET-22b (+) plasmid vector (Merck (Novagen)) (Figure S3a). The recombinant plasmid was transformed into TOP10 *E. coli* competent cells (Thermo Fisher SCIENTIFIC (Invitrogen), Waltham, MA, USA). The TOP10 *E. coli* was incubated on ice for 30 min and treated with heat-shock for 30 s at 42 °C. For recovery, 500 μL of super optimal broth with catabolite repression medium was added with gentle agitation for 1 h at 37 °C. The recombinant plasmid-transformed TOP10 *E. coli* was maintained onto Luria–Bertani (LB) agarose media corresponding ampicillin, an antibiotic, for clone selection. The appropriate colony harboring the recombinant plasmid selected by antibiotic screening was incubated in LB liquid media with agitation for 12–16 h at 37 °C. The recombinant plasmids were extracted by QIAquick Gel Extraction Kit (QIAGEN, Venlo, The Netherland) according to the manual and transformed into BL21 *E. coli* competent cells (BioDynamics Laboratory Inc., Tokyo, Japan) for protein expression. After the BL21 *E. coli* was plated onto LB agarose media overnight, appropriate colony harboring the recombinant plasmid was incubated in LB media with agitation until OD600 reached to a value of 0.6. Then, isopropyl-β-d-thiogalactoside (Nacalai Tesque, Kyoto, Japan) was added for inducing protein expression and incubated further for 2 h. All the samples were precipitated by centrifugation (2580 g, 20 min, 4 °C) and lysed by sonication in Tris-HCl buffer (20 mM Tris) adjusted to pH 7.9 with 1 N HCl. The pellets were collected by centrifugation (21,100× *g*, 20 min, 4 °C) and lysed with pipetting in Equilibration buffer (5 mM imidazole, 500 mM NaCl, 20 mM Tris, 8 M urea) adjusted to pH 7.9 on ice. The lysed sample was centrifuged (21,100× *g*, 20 min, 4 °C) and the supernatant was filtered through a 0.45 μm of filter membrane to remove the debris. The sample was purified by His60 Ni Superflow Resin and Gravity Column (Clontech Laboratories, Inc., Mountain View, CA, USA) according to the manual. The sample was agitated in the column for 1 h at 4 °C to let His-tagged target protein to bind to His60 Ni resin. After the column was washed with Equilibration buffer followed by Wash buffer (60 mM imidazole, 500 mM NaCl, 20 mM Tris, 8 M urea) adjusted to pH 7.9, the target protein was eluted with Elution buffer (1 M imidazole, 500 mM NaCl, 20 mM Tris, 8 M urea) adjusted to pH 7.9. The protein sample was separated by dialysis with PlusOne Mini Dialysis Kit (GE Healthcare, Chicago, IL, USA) in phosphate buffered saline (PBS) containing l-arginine (500 mM). The recombinant POSTN was validated by western blotting as an 85–90 kDa protein, which corresponds to the predicted molecular weight (Figure S3b,c).

### 4.3. Animals

All animal care and treatments were conducted in accordance with the National Institutes of Health Guide for the Care and Use of Laboratory Animals and the guidelines of the Kitasato University. All animal studies were approved by the President of Kitasato University through the judgment by Institutional Animal Care and Use Committee of Kitasato University (Approval numbers 16-043 (5 December 2016), 17-085 (21 April 2017) and 18-022 (27 June 2018)). Four to six-week-old male Wistar rats (Clea Japan, Inc., Tokyo, Japan) were maintained with a standard laboratory diet and tap water and exposed to a 12 h/12 h light-dark cycle at 22 ± 5 °C.

### 4.4. MCT-Induced PAH Model Rats

MCT-induced PAH model rats were produced as described previously [50]. Briefly, MCT dissolved in 1 N HCl was neutralized to pH 7–8 with 1 N NaOH and diluted to 24 mg/mL with saline. Four-week-old male Wistar rats received a single MCT (60 mg/kg) injection via intraperitoneal cavity. CONT rats received an equal volume of saline injection. Two or three weeks after the injection, RVs were harvested from the rats and used for the following experiments.

### 4.5. Cell Culture

#### 4.5.1. Isolation of RVFbs

RVFbs were isolated from RVs of MCT (3 weeks)-injected rats and CONT rats, and also from non-treated normal rats as described previously [23]. The heart excised from rats was enzymatically digested by 0.02% collagenase using a modified Langendorff apparatus. The isolated RVs were minced and suspended in Dulbecco Modified Eagle’s Medium (DMEM) (Wako Pure Chemical Industries, Ltd.). After the centrifugation (360× *g*, 3 min, 4 °C), the precipitated cells were washed and seeded on a 100 mm-culture dish in DMEM containing 10% fetal bovine serum (FBS) and 1% antibiotic-antimycotic mixed solution (Nacalai Tesque). Ninety-minutes after incubation (37 °C, 5% CO_2_), non-adherent cells were washed-out. The remaining cells were further cultured as RVFbs in DMEM containing 10% FBS.

#### 4.5.2. Cell Culture

RVFbs (passage 1–5) and H9c2 cardiomyoblasts (passage 16–28, ATCC, Manassas, VA, USA) were cultured in DMEM supplemented with 10% FBS and 1% antibiotic-antimycotic mix stock solution as previously described [23,50]. Cells at confluence were peeled off with Trypsin- ethylenediaminetetraacetic acid (EDTA) (Nacalai Tesque), suspended in culture medium and seeded in 6- or 12-well culture dish for experiments. RVFbs isolated from non-treated normal rats were starved in DMEM without serum for 24 h before stimulation. The starved RVFbs were stimulated with recombinant rat POSTN (100–1000 ng/mL) or PBS (solvent) in the presence or absence of pretreatment with BAY11-7082, PD98059 or SP600125 for 30 min. Then, they were used for the following experiments.

### 4.6. Western Blotting

Western blotting was performed as described previously [23]. The protein of RV tissues or RVFbs was lysed in Triton-based lysis buffer (1% Triton X-100, 20 mM Tris, 150 mM NaCl, 1 mM EDTA, 1 mM ethylene glycol tetraacetic acid, 2.5 mM sodium pyrophosphate, 1 mM β-glycerol phosphate, 1 mM NA_3_VO_4_, 1 μg/mL leupeptin, and 0.1% protease inhibitor cocktail (Nacalai Tesque)). The protein concentration of lysate was determined using the bicinchoninic acid method (Pierce, Rockford, IL, USA). Equal amounts of proteins (10 μg) were separated by sodium dodecyl sulphate-polyacrylamide gel electrophoresis and transferred to a nitrocellulose membrane (Pall Corporation, Washington, NY, USA). After blocking with 0.5% skim milk (for total proteins) or 3% bovine serum albumin (for phosphorylated proteins), the membranes were incubated with primary antibody at overnight at 4 °C. After incubating the membrane with HRP-conjugated secondary antibody for 1 h, the chemiluminescence signal was detected by using an EZ-ECL Kit (Biological Industries, Kibbutz Beit Haemek, Israel) and ATTO light capture system (AE-6972, ATTO, Tokyo, Japan), which was analyzed by using CS analyzer 3.0 software (ATTO).

### 4.7. Histology

Immunohistochemical staining was performed as described previously [50]. RVs isolated from the hearts of MCT (3 weeks)-injected or CONT rats were fixed in 10% neutral buffered formalin (Wako Pure Chemical Industries, Ltd.), dehydrated and used for histological analysis. Fixed RVs were embedded in paraffin, and thin tissue sections (4 μm) were made. After deparaffinized by the standard procedure, immunostaining against anti-POSTN or anti-iNOS antibody was performed using a Dako LSAB2 Kit/HRP (DAB, Dako, Glostrup, Denmark). Images were obtained with a full high definition HDMI camera (TrueChrome II Plus, Tucsen, Fuzhou, Fujian, China)-equipped fluorescence microscope (BX-51, OLYMPUS, Tokyo, Japan).

### 4.8. NO Measurement

NO produced by RVFbs was measured using an NO-specific fluorescent dye 4,5-diamino- fluorescein (DAF-2, Sekisuimedical, Tokyo, Japan) as described previously [51]. Briefly, RVFbs stimulated with recombinant rat POSTN or solvent for 24 h were treated with DAF-2 (100 nM) in physiological salt solution (PSS) for 10 min at 37 °C in the presence or absence of l-NAME (600 μM). After 200 μL of PSS was collected as negative control, l-arginine (1 mM) was added in the presence or absence of SNP (100 μM) for 120 min. The fluorescence of collected PSS at excitation 485 nm and emission 535 nm was measured by using a microplate reader (TriStar LB941; Berthold Technologies, Bad Wildbad, Germany).

### 4.9. Measurement of Ca^2+^ Inflow through LTCC

Ca^2+^ inflow through LTCC was examined by a measurement of intracellular calcium concentration using Fura-2 AM (Biotium Inc, Chemie Brunschwig AG, Basel, Switzerland), a calcium indicator, as described previously [23]. H9c2 cardiomyoblasts grown to confluence on a coverslip were incubated with normal 4-(2-Hydroxyethyl)-1-piperazineethanesulfonic acid (HEPES)-Tyrode solution (1.8 mM CaCl_2_, 143 mM NaCl, 5.4 mM KCl, 0.33 mM NaH_2_PO_4_ 2H_2_O, 0.5 mM MgCl_2_ 6H_2_O, 5.5 mM Glucose and 5 mM HEPES) containing Fura-2 AM (5 μM) for 30 min at 37 °C. Then, the cells were alternately excited at 340 and 380 nm by using a rotating filter wheel, and the fluorescence (emissions at 500 nm) of Fura-2 AM was obtained by a dual-wavelength fluorometer (CAM-230, Japan Spectroscopic Co, Ltd., Tokyo, Japan). Ca^2+^ inflow through LTCC was induced by KCl (30 mM). F340/F380 ratio (F), an indicator of [Ca^2+^]*_i_*, was calculated and normalized by the basal fluorescence (F_0_) at 30 s before KCl was added.

### 4.10. Statistical Analysis

Data are presented as means ± standard error of the mean (S.E.M.). In two-group comparison, statistical analyses were performed using *t*-test. In multi-group comparison, statistical analyses were performed by one-way or two-way ANOVA followed by *Bonferroni’s* post hoc test. A value of *p* < 0.05 was considered statistically significant.

## 5. Conclusions

In conclusion, we for the first time demonstrated that POSTN enhances iNOS expression and subsequent NO production via ERK1/2, JNK and NF-κB signaling pathways in RVFbs. The enhanced NO production in RVFbs might cause systolic dysfunction via the suppression of LTCC activity of cardiomyocytes in PAH-induced RVF. These new findings suggest that POSTN is an attractive therapeutic target for PAH-induced RVF.

## Supplementary Materials

Supplementary materials can be found at http://www.mdpi.com/1422-0067/20/1/62/s1.
