# Supplementary material for: Periostin Mediates Right Ventricular Failure through Induction of Inducible Nitric Oxide Synthase Expression in Right Ventricular Fibroblasts from Monocrotaline-Induced Pulmonary Arterial Hypertensive Rats
Source: Int J Mol Sci. 2018 Dec 24;20(1):62. doi: 10.3390/ijms20010062 (PMC6337160; doi:10.3390/ijms20010062)
Supplement: Supplementary file 1 [file ijms-20-00062-s001.pdf]

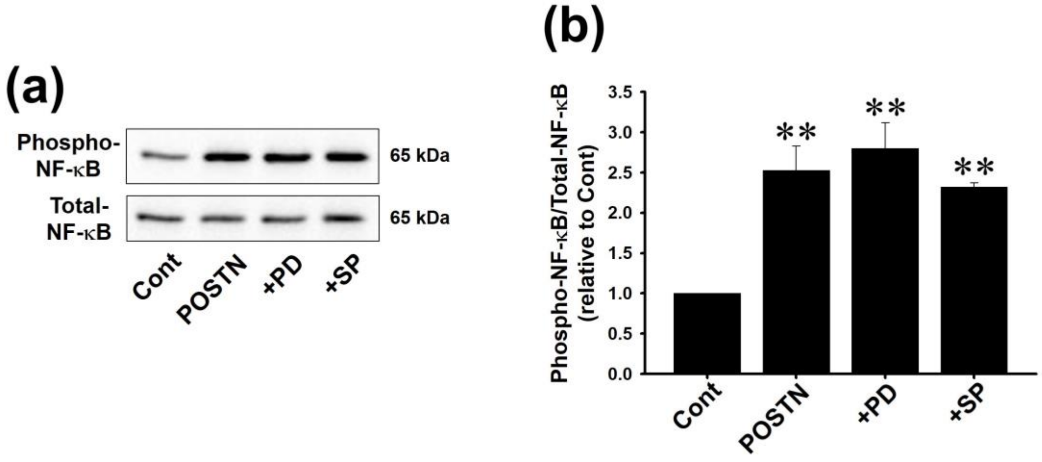

Figure S1. The pharmacological inhibition of extracellular signal-regulated kinase (ERK) or c-Jun N-terminal kinase (JNK) signaling pathway had no influence on the periostin (POSTN)-induced activation of nuclear factor-kappa B (NF-κB). Ritht ventricular fibroblasts (RVFbs) were stimulated with recombinant rat POSTN (1000 ng/ml) in the presence or absence of pretreatment with inhibitor of mitogen-activated protein kinase kinase/ERK signaling pathway [PD98059 (+PD, 50 μM)] or JNK [SP600125 (+SP, 10 μM)] for 30 min or solvent (Cont) for 1 h. Phosphorylation levels of NF-κB in RVFbs was evaluated by Western blotting. (a) Representative blots were shown. (b) Phosphrylation level of NF-κB was corrected by total-NF-κB, and the normalized phosphorylation relative to Cont was shown as mean ± standar error of the mean (S.E.M.) (n=4). ** $p<0.01$ vs. Cont.

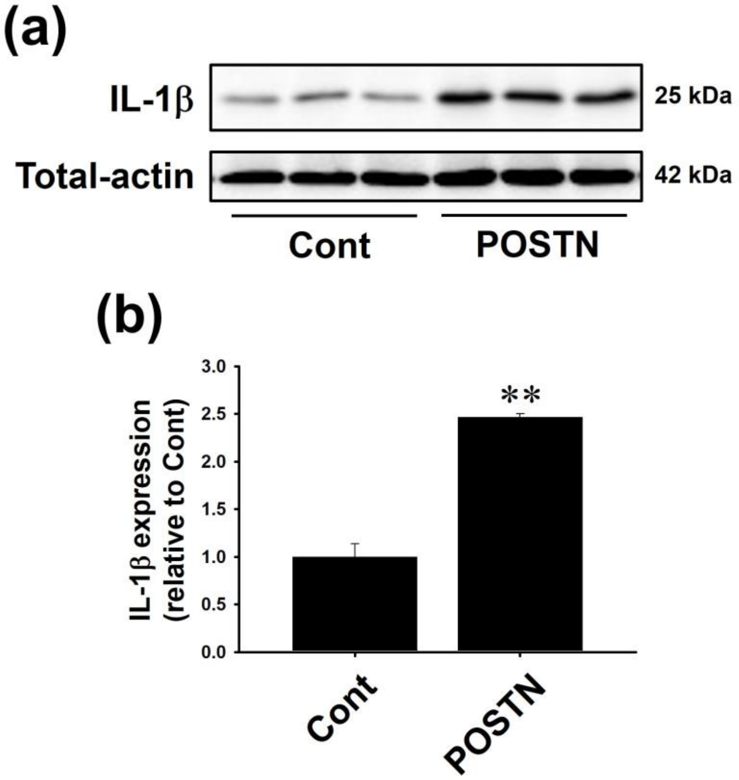

Figure S2. Recombinant rat POSTN enhanced the expression of interleukin (IL)-1β in RVFbs. RVFbs were stimulated with recombinant rat POSTN (1000 ng/ml) or solvent (Cont) for 24 h. IL-1β expression in RVFbs was evaluated by Western blotting. (a) Representative blots were shown. (b) Expression level of IL-1β was corrected by total-actin, and the normalized expression relative to Cont was shown as mean ± S.E.M. (n=3). ** $p<0.01$ vs. Cont.

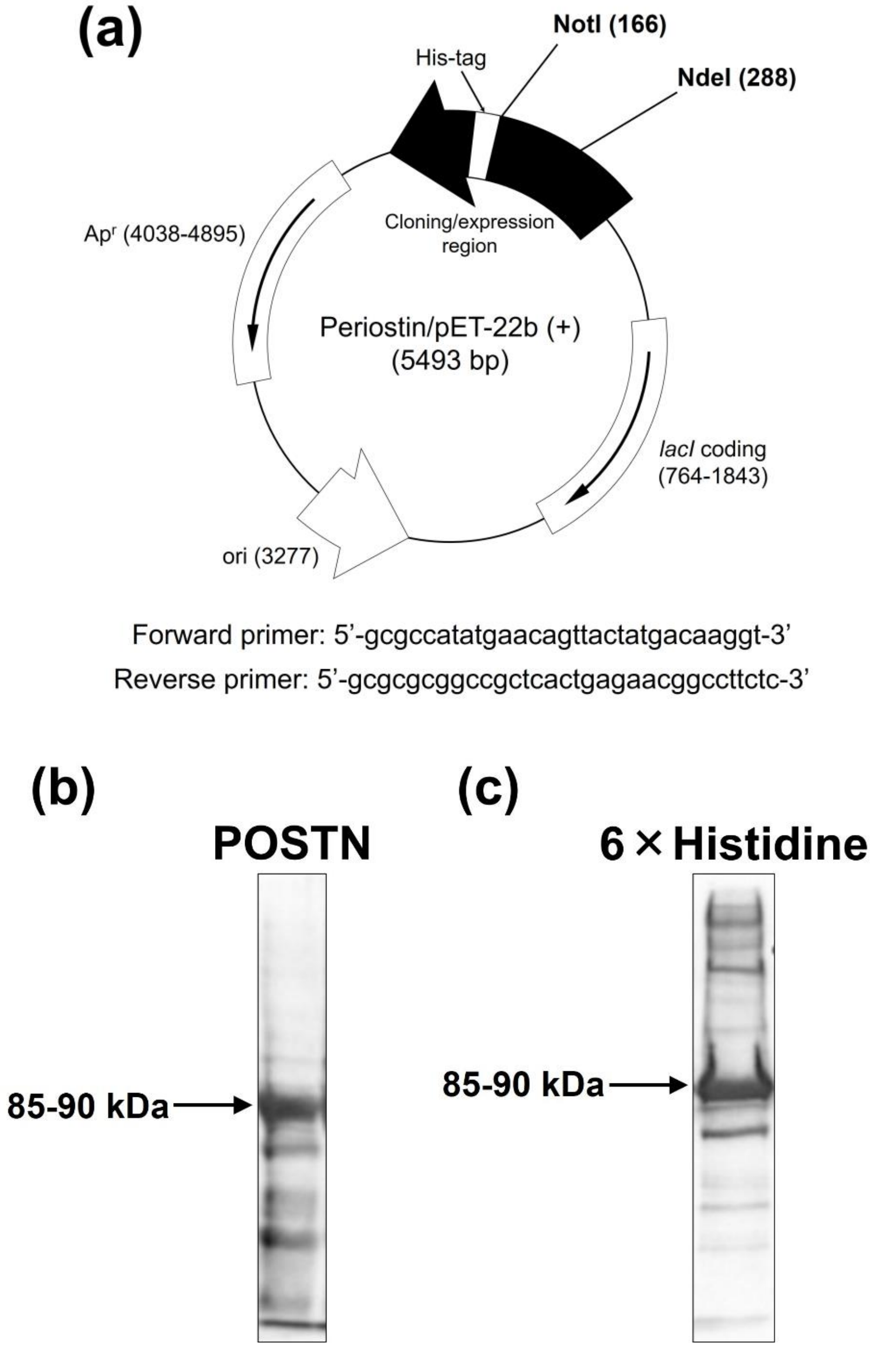

Figure S3. Production of recombinant rat POSTN protein in *Echerichia coli*. (a) The gene of full-length rat POSTN was amplified by reverse transcript-polymerase chain reaction using forward and reverse primer containing recognition sequences of NdeI or NotI, respectively, in 5′ ends. The resulting POSTN cDNA fragment was digested with NdeI and NotI, and ligated into predigested pET-22b (+) plasmid vector. Ap^{r}: antimicrobial resistance, ori: replication origin, *lacI*: lac repressor. The produced recombinant POSTN protein fused to 6xHistidine (His-tag) was validated by Western blotting. The recombinant protein was separated by sodium dodecyl sulfate polyacrylamide gel electrophoresis, and transferred to a nitrocellulose membrane. Representative blots using anti-POSTN antibody (b; Proteintech, Rosemont, IL, USA) and anti-6xHistidine antibody (c; Wako Pure Chemical Industries, Ltd, Osaka, Japan) were shown. Bands were detected in predicted size of POSTN at 85-90 kDa.
